# Usutu Virus Infects Human Placental Explants and Induces Congenital Defects in Mice

**DOI:** 10.3390/v14081619

**Published:** 2022-07-25

**Authors:** Hélène Martin, Jonathan Barthelemy, Yamileth Chin, Mathilde Bergamelli, Nathalie Moinard, Géraldine Cartron, Yann Tanguy Le Gac, Cécile E. Malnou, Yannick Simonin

**Affiliations:** 1 Institut Toulousain des Maladies Infectieuses et Inflammatoires (Infinity), Université de Toulouse, INSERM, CNRS, UPS, Toulouse, France; helene.martin@inserm.fr (H.M.); yamileth.chin-acosta@inserm.fr (Y.C.); mathilde.bergamelli@gmail.com (M.B.); 2 Pathogenesis and Control of Chronic and Emerging Infections, University of Montpellier, INSERM, EFS, Montpellier, France; jonathan.barthelemy@inserm.fr; 3 Instituto Conmemorativo Gorgas de Estudios de la Salud, Ciudad de Panamá, Panamá; 4 Développement Embryonnaire, Fertilité, Environnement (DEFE), INSERM UMR 1203, Université de Toulouse et Université de Montpellier, France; moinard.n@chu-toulouse.fr; 5 CECOS, Groupe d’Activité de Médecine de la Reproduction, CHU Toulouse, Hôpital Paule de Viguier, Toulouse, France; 6 CHU Toulouse, Hôpital Paule de Viguier, Service de Gynécologie Obstétrique, Toulouse, France; cartron.g@chu-toulouse.fr (G.C.); tanguylegac.y@chu-toulouse.fr (Y.T.L.G.)

**Keywords:** arbovirus, flavivirus, Usutu virus, placenta, vertical transmission

## Abstract

Usutu virus (USUV) is a neurotropic mosquito-borne flavivirus that has dispersed quickly in Europe these past years. This arbovirus mainly follows an enzootic cycle involving mosquitoes and birds, but can also infect other mammals, causing notably sporadic cases in humans. Although it is mainly asymptomatic or responsible for mild clinical symptoms, USUV has been associated with neurological disorders, such as encephalitis and meningoencephalitis, highlighting the potential health threat of this virus. Among the different transmission routes described for other flaviviruses, the capacity for some of them to be transmitted vertically has been demonstrated, notably for Zika virus or West Nile virus, which are closely related to USUV. To evaluate the ability of USUV to replicate in the placenta and gain access to the fetus, we combined the use of several trophoblast model cell lines, ex vivo human placental explant cultures from first and third trimester of pregnancy, and in vivo USUV-infected pregnant mice. Our data demonstrate that human placental cells and tissues are permissive to USUV replication, and suggest that viral transmission can occur in mice during gestation. Hence, our observations suggest that USUV could be efficiently transmitted by the vertical route.

## 1. Introduction

The arboviral risk is globally rising on the European continent and more generally worldwide, and it is already well established in various regions, particularly in Africa and Latin America. Among potential emerging viruses, Usutu virus (USUV) has drawn the attention of the scientific community in recent years, since this virus has massively dispersed out of Africa, mainly into Europe [1]. USUV is an icosahedral-enveloped virus of approximately 40–60 nm in diameter. Its genome is a single-stranded positive-sense RNA of 11 kb in length, with a 5′ cap structure and one large open reading frame encoding a unique polyprotein of 3434 amino acids. This polyprotein is post-translationally processed into three structural proteins: capsid, pre-membrane/membrane, and envelope (Env), and eight non-structural proteins (NS1, NS2a, NS2b, NS3, 2K, NS4a, NS4b, and NS5) [2]. USUV strains are classified into eight genetic lineages divided in two major African or European groups: Africa 1, 2, and 3, and Europe 1, 2, 3, and 4, that have all been detected in Europe, except for Africa 1 [3].

USUV was first identified in 1959 in South Africa from a mosquito of the *Culex neavei* species, and isolated by intracerebral inoculation of newborn mice [4,5,6,7]. Similar to Zika virus (ZIKV) and West Nile virus (WNV), with which it shares many common features, USUV belongs to the genus *Flavivirus* from the *Flaviviridae* family. It is maintained through an enzootic cycle involving birds (mainly passerine and Strigiformes) acting as amplifying hosts, and ornithophilic mosquitoes, such as *Culex pipens*, as vectors [8]. Humans and other mammals, such as horses, dogs, rodents, and wild boars, are considered as accidental hosts [8]. In humans, USUV infection was first described in Africa, in Central African Republic (1981), and in Burkina Faso (2004) [9]. Major USUV epizootics affecting avifauna, associated with a large epidemic of West Nile virus (WNV), were demonstrated in Europe in 2016 and in 2018 [8]. Molecular and serologic evidence of USUV infection in European blood donors suggests a silent spread of this virus among asymptomatic humans, which could thus be a concern for blood transfusions or organ transplants [8]. In Europe, epizootics were accompanied by several descriptions of human neurological disorders, including facial paralysis, encephalitis, meningitis, and meningoencephalitis, in immunocompromised and immunocompetent patients [10,11,12,13,14,15,16,17,18,19,20,21,22]. USUV infection had been reported in a dozen of European countries, and to date, more than one hundred cases of acute human infection have been described, mainly in Europe [8].

Despite the fact that USUV is an emerging pathogen dispersed quickly in Europe, very little is known about its pathogenicity and transmission routes. Among the different transmission routes described for arboviruses, it is known that some flaviviruses, such as ZIKV and WNV, have the ability to be vertically transmitted [23,24,25]. Vertical transmission of ZIKV was at the heart of concerns during the major epidemic of 2015–2016 in Brazil and Latin America, with the discovery of congenital Zika syndrome (CZS) observed in newborns of mothers who contracted the virus during their pregnancy. CZS is mainly characterized by microcephaly, associated with severe cerebral malformations and ocular alterations, among others [26,27]. In recent years, the maternal-fetal transmission of ZIKV has been the subject of numerous studies seeking to characterize the cells and mechanisms allowing the transplacental passage of the virus towards the fetal compartment [25,28]. ZIKV has been shown to infect a wide variety of cell types at the maternal–fetal interface, including decidual cells, endothelial cells, cytotrophoblasts, or macrophage Hofbauer cells [29,30,31,32,33]. Moreover, placental cell permissiveness has been shown to differ among cell type and gestational age, with modulation of viral receptor expression and a lower innate immune response in first-trimester versus third-trimester placenta [30,31,32,33]. This correlated with the degree of severity of the sequelae observed during ZIKV infection of animal models [34,35] or upon natural infection in human population, the earliest infection being the most severe [36].

In the present study, we examined whether USUV may be vertically transmitted via replication in the placenta. To this end, we combined the use of several trophoblast cell lines, ex vivo human placental histocultures from the first and third trimester of pregnancy, and in vivo USUV-infected pregnant mice, to assess the ability of USUV to replicate in the placenta and gain access to the fetus. Our data demonstrate that placental cells and tissues are permissive for USUV replication, and suggest that viral transmission can occur in offspring during pregnancy.

## 2. Materials and Methods

### 2.1. Ethics Statement

The biological resource center Germethèque (BB-0033-00081; declaration: DC-2014-2202; authorization: AC-2015-2350) obtained the written consent from each patient (CPP.2.15.27) for the use of human samples and their associated data. The steering committee of Germethèque gave its approval for the realization of this study on 5 February 2019. The hosting request made to Germethèque bears the number 20,190,201 and its contract is referenced under the number 19 155C.

Mice were bred and maintained according to the French Ministry of Agriculture and European institutional guidelines (appendix A STE n° 123). Experiments were performed according to national regulations and approved by the regional ethics committee of Languedoc-Roussillon (Comité Régional d’Ethique sur l’Expérimentation Animale- Languedoc-Roussillon), France (approval n° 6773-201609161356607).

### 2.2. Cell Lines

JAR choriocarcinoma cells (ATCC HTB-144), deriving from a trophoblastic tumor of the placenta, and Vero cells (ATCC CCL-81) were cultured at 37 °C and 5% CO_2_ in DMEM (Gibco), supplemented with 10% heat-inactivated fetal bovine serum (FBS, Sigma-Aldrich), 100 U/mL penicillin—100 µg/mL streptomycin (Gibco), and 100 µg/mL normocin (Invivogen). HIPECs (human invasive proliferative extravillous cytotrophoblast) [37], an immortalized cell line deriving from first-trimester primary cytotrophoblasts, obtained from Dr T. Fournier (Inserm, Paris; Transfer agreement n° 170448), were cultured in DMEM/F12 medium (Gibco) at 50/50 ratio (*v*/*v*) and supplemented as above.

### 2.3. USUV Strains, Viral Stock Production and Cell Infection

USUV (Africa 2 strain, Rhône 2705/France/2015-KX601692) was provided by Anses (Agence nationale de sécurité sanitaire de l’alimentation, de l’environnement et du travail) and was not propagated more than three times on Vero cells. Viral stocks were prepared by infecting sub-confluent Vero cells at a multiplicity of infection (MOI) of 0.01 in DMEM with 2% heat-inactivated FBS. Cell supernatant was then collected between 5 to 7 days post-infection and harvested after centrifugation at 300× *g* to remove cellular debris. Viral titers were determined by the 50% tissue culture infective dose (TCID50) using the Spearman–Kärber method, and were expressed as TCID50 per mL [38].

For infection, cells at 60–70% confluence were rinsed once with phosphate-buffered saline (PBS), and USUV diluted to the required MOI were added to the cells in a low medium volume. Cells were incubated for 2 h at 37 °C and then culture medium was added to each well. As a control, cells were incubated with the culture supernatant from Vero cells (mock condition).

### 2.4. Placental Histocultures

Placental histocultures were realized as described [39] on first-trimester placentas (6 placentas; mean = 10.65 ± 0.35 (SEM) weeks of amenorrhea, i.e., 9.65 ± 0.35 weeks of pregnancy; age of the women: mean = 27 ± 2.3 years old) or term placentas (3 placentas; mean = 40.95 ± 0.05 weeks of amenorrhea, i.e., 38.95 ± 0.05 weeks of pregnancy; age of the women: mean = 32.33 ± 4.9 years old). Trophoblastic villi were dissected in small explants and infected or not by USUV viral stock diluted 1:1 with DMEM medium in 1 mL final volume overnight before deposition on gelatin sponges (Gelfoam, Pfizer) upon intensive washes in PBS. Culture medium was collected and renewed every 3 to 4 days. At 15 days of culture, placental explants were fixed or flash frozen for future use.

### 2.5. Mouse Experiments

C57BL/6 WT pregnant mice were purchased from Janvier Laboratories (Saint-Berthevin Cedex, France). Mice were inoculated with USUV by subcutaneous (footpad) route with 10^4^ TCID50 in 50 µL of PBS on embryonic days 6 (E6) or E12 and sacrificed on E13 or after the birth, respectively. Mice were infected at ECE (Etablissement Confiné d’Expérimentation), a level 3 animal facility of the University of Montpellier. USUV-infected mice and control mice were euthanized by cervical dislocation or with a lethal dose of pentobarbital (Sigma-Aldrich, Darmstadt, Germany) at indicated day post-infection, depending on the experimental design. Organs and tissues were snap frozen with liquid nitrogen for viral burden.

### 2.6. Immunofluorescence

Immunofluorescence against anti-flavivirus group antigen were realized as described [40] using mouse 4G2 primary antibody (Novus Biologicals) diluted 1:400. Widefield acquisitions were realized using Apotome microscope (Zeiss) and image processing was performed using ImageJ.

### 2.7. Intracellular Staining and Flow Cytometry Analysis

Cells were fixed and permeabilized with BD Cytofix/Cytoperm fixation/permeabilization buffer according to the manufacturer’s instructions. Envelope antigen was then detected with a 1:400 dilution of Alexa fluor 488-conjugated mouse monoclonal antibody 4G2 (Novus Biologicals) for 25 min at 4 °C. Cell fluorescence was then analyzed on a Macsquant VYB Flow Cytometer (Miltenyi Biotec), by using FCS and FITC fluorescence parameters, and by subtracting cell autofluorescence background. Data were analyzed with FlowJo (BD) software.

### 2.8. Analysis of Cell Growth and Viability

10^5^ cells were initially seeded in 12-well plates and infected or not by USUV at MOI 3 during 2 h, before medium change for removing of viral inoculum. At different times post-infection, supernatants were collected, and cells were trypsinized. Number of viable cells was determined upon trypan blue staining after trypsinization. Number of dead cells was determined by addition of previously determined adherent dead cells and floating dead cells, collected in supernatant in a given time point, and expressed as percentage of the total cell number.

### 2.9. TUNEL Assay

TUNEL assay was done using Click-iT Plus TUNEL Assay for In Situ Apoptosis Detection Kit (Life Technologies), following manufacturer’s instructions, as previously described [39]. Image acquisition was performed on a Zeiss Axiovert 200 microscope.

### 2.10. RNA Extraction

Total RNA was extracted from cells using RNeasy Plus Mini Kit (Qiagen), and from placental explants using miRNeasy Mini Kit (Qiagen), according to the manufacturer’s instructions. When extracted from cell or tissue supernatant, RNA extraction was realized using QiaAmp Viral RNA Mini Kit (Qiagen) from 220 µL supernatant. Upon extraction, RNA concentration and quality were systematically determined using a NanoDrop spectrophotometer.

### 2.11. RT-qPCR Analysis

Upon extraction, 500 ng RNA were subjected to reverse transcription reaction using LunaScript RT SuperMix Kit (New England Biolabs), following manufacturer’s protocol. cDNA was then used as matrix in qPCR reaction using Sybr Green I Master Mix (Roche) and the following primers: USUV-Forward: AACAGACGGTGATGCGAACT; USUV-Reverse: TACAGCTTCGGAAACGGCTT; β-actin-Forward: GTGCTGTCCCTGTACGCCTCT; and β-actin-Reverse: GGCCGTGGTGGTGAAGCTGTA. qPCR reactions were carried out with a Roche LightCycler 480 apparatus using the following program: 95 °C for 5 min, then 40 cycles of 95 °C for 15 s, and 60 °C for 10 sec. The fold induction in viral transcript expression was quantified by calculating the 2^−ΔΔCT^ value, with β-actin mRNA as internal control. For quantification of viral transcript release in cell or tissue supernatant, qPCR reactions were performed with primers targeting USUV transcripts only. Results were compared to those obtained with a standard curve realized using a plasmid containing the USUV cDNA with known concentration and genome copy correspondence.

### 2.12. RT2 Profiler PCR Arrays

Upon RNA extraction, 500 ng of RNA were subjected to reverse transcription using RT2 First Strand kit (Qiagen), following manufacturer’s protocol. The resulting cDNAs were then used for RT2 profiler PCR arrays (Human Antiviral Response PAHS-122Z, Qiagen), for profiling 84 genes specific for innate immune response, with five housekeeping genes as internal controls (ACTB, B2M, GAPDH, HPRT1, and RPLP0), following the manufacturer’s instructions. qPCR reactions were carried out with a Roche LightCycler 480 apparatus using the following program: 95 °C for 10 min, then 45 cycles of 95 °C for 15 s, and 60 °C for 1 min, followed by a melting curve acquisition step. The fold change of gene expression and Student’s *t*-test were automatically calculated by the RT2 profiler RT-PCR array data analysis software, version 5.1.

### 2.13. Histology and Immunohistochemistry

For histology studies of placental tissues, explants were fixed 24 h in 10% formalin, dehydrated, embedded in paraffin, sectioned (5 μm), and de-waxed. Upon epitope retrieval, immunohistochemistry was carried out as described using mouse anti-placental alkaline phosphatase (Biolegend; 1 μg/mL) as primary antibody [39]. Image acquisition was performed on a Panoramic 250 scanner (3DHISTECH).

Alternatively, for detection of USUV Env antigen, immunohistochemistry was performed on the Discovery Ultra Automated IHC staining system using the Ventana DAB Map detection kit. For viral antigen immunostaining, antigen retrieval was performed for 4 min at 37 °C with Protease 1 solution from Ventana, which is an endopeptidase (alkaline protease) of the serine protease family. Endogenous peroxidase was blocked with Discovery Inhibitor CM for 8 min at 37 °C. The slides were incubated after rinsing at 37 °C for 32 min with a mouse anti-flavivirus group antigen monoclonal antibody (Millipore, MAB10216, 1∶800 in Dako antibody diluent with background reducing components). Signal enhancement was performed using Rabbit monoclonal to mouse IgG1 + IgG2a + IgG3 as the secondary antibody (Abcam, ab133469, 1/8000) and the Discovery DAB Rabbit HQ Kit. Slides were then counterstained with hematoxylin for 8 min and manually dehydrated before coverslips were added. Slides were treated with a Hamamatsu NanoZoomer 2.0-HT scanner by MRI platform, and images were visualized with the NDP.view 1.2.47 software.

### 2.14. Measurement of Viral Burden In Vivo

Mouse organs (brain, placenta, spleen, and mammary glands) were weighed and homogenized with zirconia beads in a Fastprep 24 apparatus (MP Biomedicals) in 250 μL PBS, and RNA was extracted using the RNeasy Mini Kit (Qiagen). Blood RNAs were extracted from 100 μL of samples, with the EZ1 apparatus running the EZ1 DSP virus kit (Qiagen). Viral USUV RNA levels were measured by a one-step quantitative reverse transcriptase PCR assay on the Light Cycler 480 (Roche) with primers, probe, and cycling conditions previously described [41]. Viral burden was expressed on a log10 scale as TCID50 equivalents per gram or ml after comparison with a standard curve produced using serial 10-fold dilutions of USUV with known viral titers.

### 2.15. Statistical Analyses

GraphPad Prism (v8) software was used to perform data statistical analysis. One-way or two-way ANOVA tests were carried out, followed by Tukey’s multiple comparison test for two-way ANOVA.

## 3. Results

### 3.1. Human Placental Cell Lines Exhibit Variable Permissiveness to USUV Replication

**Figure 1:**
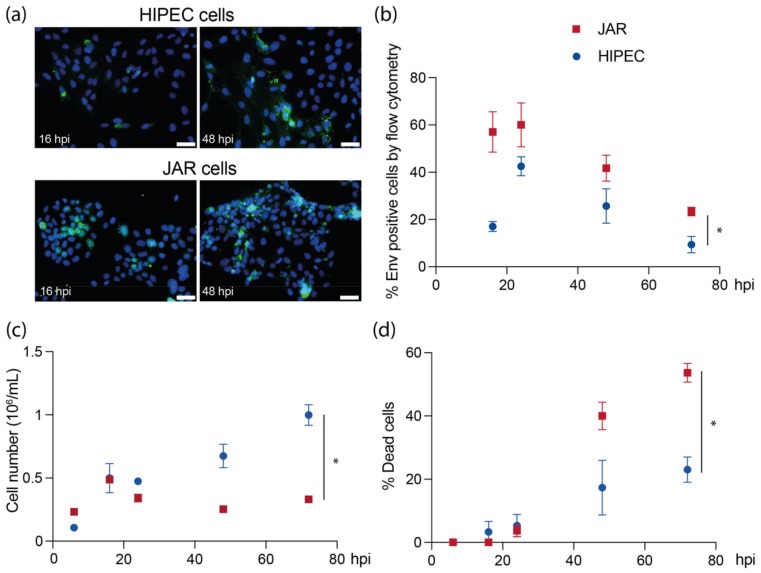
Kinetics of USUV infection in placental cell lines and effect on cell growth and viability. JAR cells or HIPECs have been infected by USUV at MOI 3 at different times before proceeding to analyses. (**a**) Indirect immunofluorescence realized against USUV Env antigen (green: Env; blue: DAPI) at 16 or 48 h post-infection (hpi). Images are representative of at least three independent experiments, and three independent fields each time. Scale bar = 50 μm. (**b**) Quantification of the percentage of infected cells determined by flow cytometry, via intracellular staining of Env antigen. (**c**) Quantification of cell growth between JAR cells and HIPECs upon USUV infection, by counting viable cell number at different time points. (**d**) Cell mortality was evaluated by trypan blue staining for JAR cells and HIPECs at different times upon USUV infection. In (**b**–**d**), symbols represent mean ± SEM for three independent experiments. In each experiment, a two-way ANOVA statistical test was performed and indicated a significant difference between cell lines (*, *p* < 0.05).

To assess the ability of USUV to replicate in human placental cells, we used two cellular models: the JAR cells, which share many characteristics of early placental trophoblasts such as the ability to differentiate into syncytiotrophoblastic cells in vitro and secrete gonadotropic hormone [42,43]; and HIPECs, deriving from first-trimester cytotrophoblasts with invasive capacities [37]. USUV was able to disseminate in both cell types (Figure 1a,b), however with different efficiencies and impact on cell growth and survival. Globally, a significant difference was observed in the level of infection between JAR cells and HIPECs. In JAR cells, a high proportion of cells, nearly 60%, were infected as soon as 16 h post-infection. In contrast, only 17% of HIPECs were infected at the same time (Figure 1b). A maximum level of infection was reached for both cell lines at 24 h before a decrease. At that time, a decrease in cell growth was observed for both HIPECs and JAR cells (Figure 1c). However, HIPECs were then able to resume their growth, while the JAR cell growth remained highly affected by USUV infection. By evaluating the mortality rate in both cell lines upon infection, we observed that the percentage of cell death was significantly higher in JAR cells than in HIPECs, reaching more than 50% comparing to less than 25%, respectively (Figure 1d). Taken together, these results indicated a difference in susceptibility for USUV infection between the two cell lines, explaining the higher infection rate and mortality observed in JAR cells compared to HIPECs.

**Figure 2:**
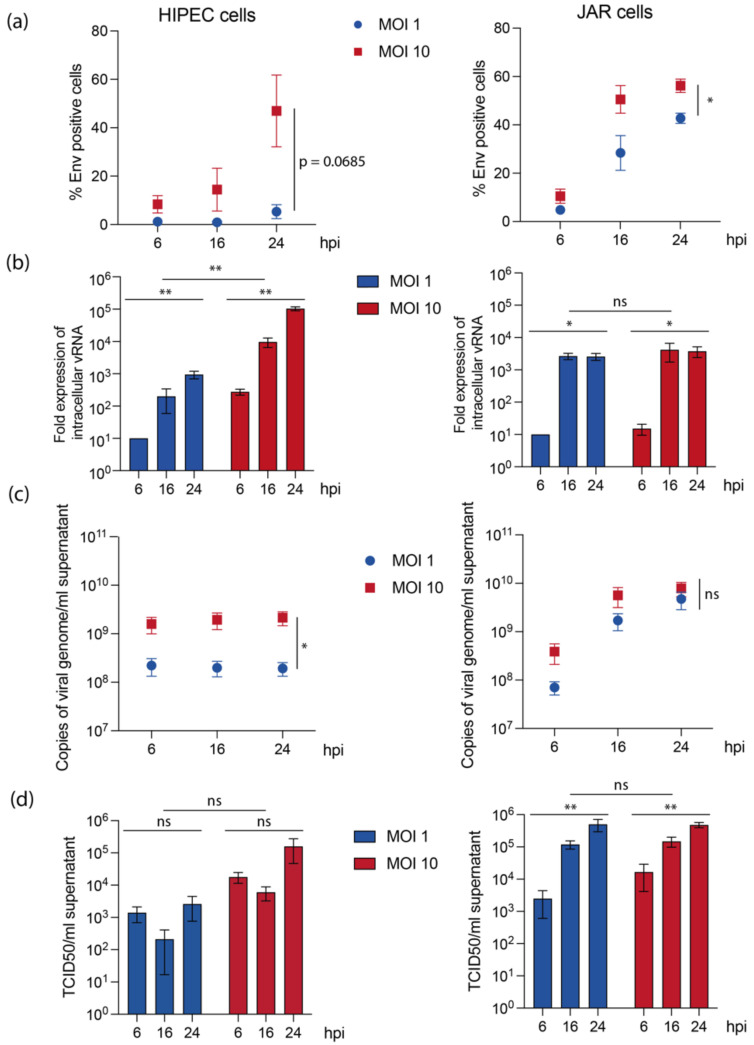
JAR cells and HIPEC allow USUV productive replication cycle with different efficiencies. HIPECs (left column) or JAR cells (right column) have been infected by USUV at MOI 1 or 10 during indicated times (hpi: hours post-infection) before proceeding to analyses. (**a**) Quantification of the proportion of infected cells determined by flow cytometry, via intracellular staining of Env antigen. Symbols represent the mean ± SEM for three independent experiments for HIPECs and four independent experiments for JAR cells. (**b**) Quantification of viral RNA extracted from infected cells, upon normalization by actin mRNA and by the level of viral RNA at 6 hpi for MOI 1. Histograms represent mean ± SEM for three independent experiments. (**c**) Quantification of viral RNA released in cell supernatant. Symbols represent the mean ± SEM for five independent experiments. (**d**) Quantification of infectious particles released in cell supernatant. Histograms represent mean ± SEM for five independent experiments. In (**a**–**d**), a two-way ANOVA statistical test was performed to evaluate a statistical difference between cell lines and MOI (ns, non-significant; *, *p* < 0.05; **, *p* < 0.005).

To address whether trophoblast cell lines may achieve productive infections, we examined different parameters during the first 24 h post-infection (Figure 2). Results obtained for infected-JAR cells confirmed their high permissiveness for USUV. Cells were highly positive for USUV Env by flow cytometry and showed high induction of USUV viral RNA (vRNA) expression (Figure 2a,b, right panels). Moreover, USUV was able to perform a productive cycle in JAR cells, as assessed by the high level of vRNA released in supernatant (Figure 2c, right panel), and by the presence of infectious particles with around 5 × 10^5^ TCID50/mL in supernatant upon 24 h post-infection (Figure 2d, right panel). Interestingly, except for flow cytometry experiments, there was no significant difference between MOI 1 or 10 in JAR cells, indicating that the maximum level of infection or viral production was already reached for MOI 1. Moreover, infection was very fast in these cells, since for most of the experiments carried out, a plateau was reached at 16 h post-infection.

Our results showed that HIPECs were also permissive for USUV replication. However, compared to JAR cells, USUV infection of HIPECs was slower and more dependent on the MOI used (Figure 2a,b, left panels). vRNA and infectious particle release in supernatant was also less efficient (Figure 2c,d, left panels), with no significant increase with time, reaching a maximum of 1.6 × 10^5^ TCID50/mL upon 24 h post-infection for MOI 10. However, despite this decrease in permissiveness compared to JAR cells, USUV was also able to productively infect HIPECs.

### 3.2. USUV Elicits a Strong Antiviral Response in JAR Cell Line

**Figure 3:**
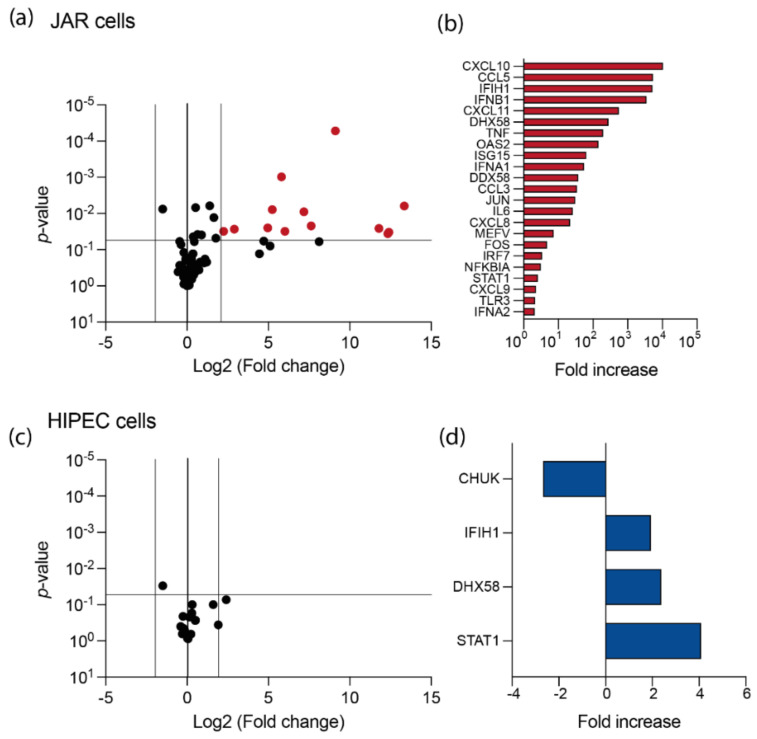
Characterization of innate immune responses of HIPEC and JAR cells upon USUV infection. (**a**) And (**c**) volcano plot representing differences in normalized mean mRNA expression in JAR cells (**a**) or HIPECs (**c**) 16 h upon USUV infection compared to non-infected cells, from three independent experiments. mRNAs exhibiting significant differences upon infection are represented by colored circles (Student’s *t*-test *p*-value ≤ 0.05 and log2 ratio ≥ 2 or ≤ −2). (**b**–**d**) Detail of the under- and over-expressed mRNAs upon USUV infection in JAR cells (**b**) or HIPECs (**d**).

To better describe the effect of USUV infection on cytotrophoblasts, the level of expression of 84 genes involved in innate antiviral response was monitored in both cell lines (Figure 3). At 16 h upon infection at MOI 10, mRNA level of numerous genes was significantly enhanced in infected JAR cells compared to non-infected cells (Figure 3a). USUV infection induced the expression of the pattern recognition receptors (PRRs) IFIH1, DDX58, and DHX58 (also known as MDA 5, RIG-I, and RIG-I-like receptor 3 (RLR-3), respectively; Figure 3b). In accordance with the stimulation of PRRs, induction of type I interferon pathway upon USUV infection, with expression of IRF7, IFNA1, and IFNB1, was also observed. In parallel with the induction of STAT1 expression, transcription of some IFN stimulated genes (ISGs) was also noticed upon USUV infection of JAR cells, such as OAS2 and ISG15. Finally, a high number of pro-inflammatory cytokines were up-regulated upon infection, such as CXCL10, CCL5, CXCL11, TNF, CCL3, CXCL8, and IL6.

In contrast to what was observed in JAR cells, innate antiviral immune response in HIPECs was quite inexistent, with few induced genes and very low amplitude of induction at the same time post-infection (Figure 3c,d). Induction of antiviral genes was no more evidenced at 24 h post-infection (data not shown), albeit the percentage of infected cells reached its maximum with around 50% of infected cells at MOI 10 (Figure 2a), indicating that this cell line was not able to elicit an efficient antiviral immune response against USUV.

### 3.3. Human Placental Tissues Are Permissive to USUV Replication

**Figure 4:**
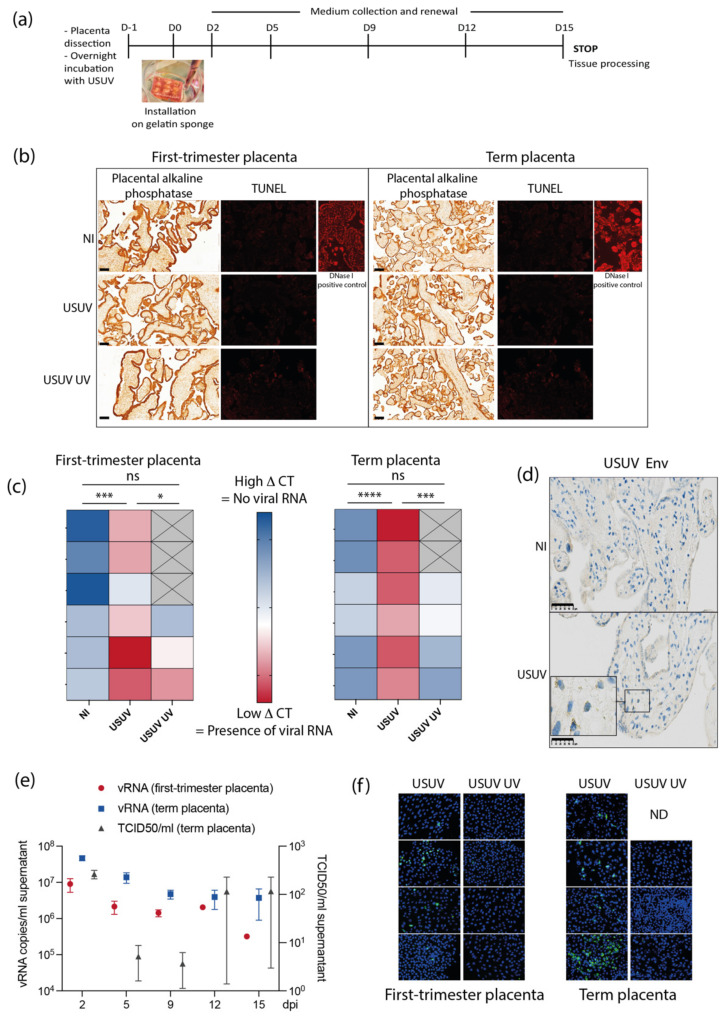
Human first- and third-trimester placentas are permissive for USUV replication. (**a**) Pipeline of placental explant infection and histoculture. (**b**) Left columns: cross sections of placental alkaline phosphatase immunohistochemistry and hematoxylin staining of placental villi from histoculture at day 15, observed by bright field microscope. Image representative from at least three independent experiments. Scale bar = 100 μm. Right columns: fluorescence-based TUNEL assay done on cross section of placental villi from histoculture at day 15. Image representative from three independent experiments (NI: non-infected; USUV UV: infection by UV-inactivated USUV). (**c**) Heat-map representing the level of viral RNA expression (normalized by actin) in placental tissues upon infection by USUV or by UV-inactivated virus (USUV UV), for six independent experiments (NI: non-infected). The ΔCT values are represented by a double gradient color map (blue: high ΔCT = no amplification of viral RNA; red: low ΔCT = amplification of viral RNA; and crossed gray: no data). A one-way ANOVA statistical test was performed to evaluate a statistical difference between conditions, followed by Tukey’s multiple comparison test (ns, non-significant; *, *p* < 0.05; ***, *p* < 0.0005; ****, *p* < 0.0001). (**d**) Anti-USUV Env immunohistochemistry done on term placental villi from histoculture at day 15. Blue staining corresponds to hematoxylin coloration of the nucleus and brown staining correspond to the USUV Env detection. Scale bar = 50 μm (NI: non-infected). (**e**) Quantification of viral RNAs (red circles: first-trimester placenta; blue squares: term placenta; and left Y-axis) and infectious particles (blue triangles, term placenta, and right Y-axis) released in supernatant by placenta histocultures at different times upon USUV infection (dpi: days post-infection). Symbols represent the mean ± SEM for three to six independent experiments. (**f**) Results of anti Env immunofluorescence realized upon reinfection of Vero cells incubated with supernatant of placental histocultures, either from first-trimester (left panels) or term (right panels) placentas, done with supernatants collected at day 15 after infection by USUV or UV inactivated USUV (ND: not determined). Blue: DAPI, green: USUV Env antigen.

To go further in the evaluation of USUV placental infection, we next used a model of human tissue explants of first-trimester or term placenta (see pipeline Figure 4a) [39,44]. First, placenta cyto-architecture and viability upon 15 days of culture were assessed by performing an anti-placental alkaline phosphatase (PLAP) immuno-histochemistry and a TUNEL assay (Figure 4b). As expected, PLAP expression was evidenced in the syncytiotrophoblast layer of the villi, with no visible alteration of the tissue architecture upon USUV infection upon 15 days of infection. Moreover, no overt cell death was observed upon infection by TUNEL assay. At 15 days post-infection, the presence of vRNAs in placental tissue was evaluated both in first-trimester and term placentas (Figure 4c). The level of vRNA in placental tissues, normalized by actin, was represented by a double gradient color heat-map, with red color indicating high amplification, and blue indicating no amplification. In both cases, presence of significant amount of vRNAs was evidenced in USUV-infected placental tissues, as indicated with red colored boxes, in comparison to non-infected tissues, or tissue infected with UV-inactivated virus, indicating that the presence of vRNAs was due to an active viral transcription process and not the remaining viral inoculum. Thus, both first- and third-trimester placental tissues allowed active transcription of USUV. Moreover, the presence of USUV Env antigen was also detected in a few cells in some, but not all, term placental explants by immuno-histochemistry (Figure 4d). We next examined vRNAs and infectious particles released in supernatant of placental histocultures in collected media along the different times post-infection. vRNAs were detected in first-trimester and term placental explant supernatant along the duration of the culture, albeit with lower quantities for first-trimester placental explants (Figure 4e, red and blue dots). Release of infectious viral particles was also monitored by TCID50 titration on histoculture supernatants. No infectious particles could be detected in supernatant of first-trimester explants (data not shown) in contrast to term explants, in which infected cells were sometimes detected (Figure 4e, grey dots). Finally, the different histoculture supernatants at day 15 post-infection were incubated with Vero cells during 24 h, and an indirect immunofluorescence against USUV Env was realized (Figure 4f). Infected Vero cells were observed with supernatants from first-trimester and term placenta histocultures, and high productive infection was recorded in some of them, confirming the results obtained in Figure 4e and the intrinsic variability of the placenta permissiveness.

### 3.4. USUV Can Achieve Congenital Infection in Immunocompetent Mice and Causes Occasional Fetal Demise

To the best of our knowledge, the capacity of USUV to infect fetuses has not been studied in any experimental model. We chose to study the potential of USUV to be transmitted vertically in vivo using immunocompetent mice. Individual fetuses were evaluated morphologically for size and appearance and for the presence of virus in the brain and/or blood. A low intrauterine transmission rate was observed in pups born to immunocompetent mice infected in the first week of gestation whereas no transmission was detected when mice were infected in the second week (Table 1).

**Figure 5:**
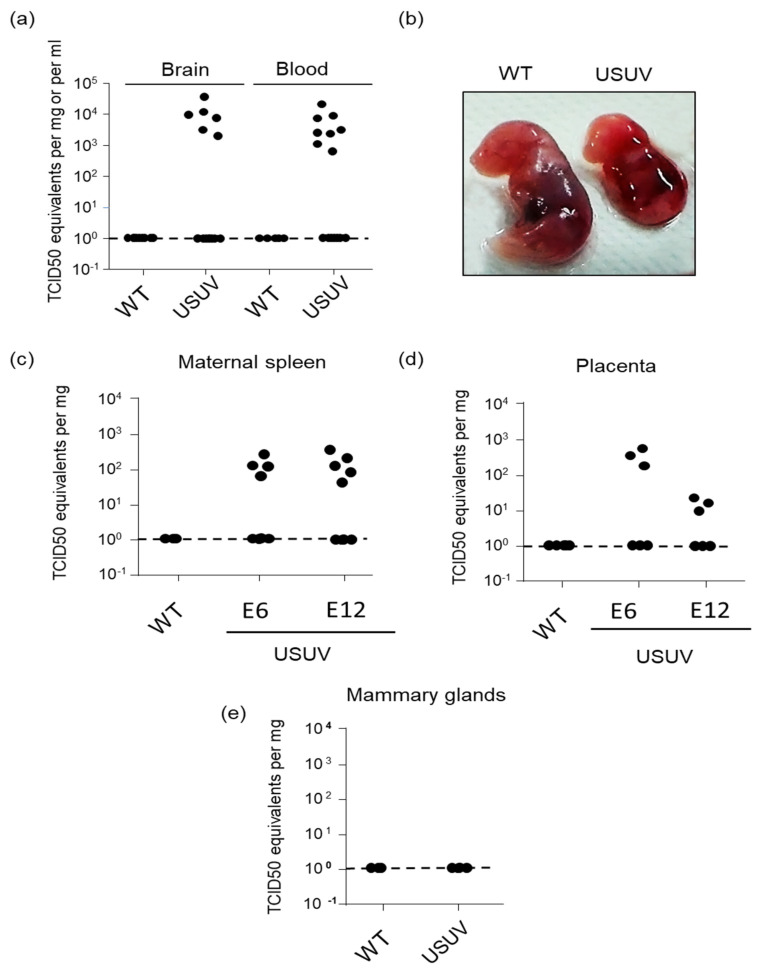
USUV can achieve congenital infection of immunocompetent mice and causes occasional fetal demise. C57BL/6 pregnant mice were inoculated with USUV by subcutaneous (footpad) route with 104 TCID50 in 50 µL of PBS. (**a**) Brain and blood of suckling mice were collected after the birth for the brain and 2 weeks later for blood. Viral burden was measured by RT-qPCR assay and indicated by TCID50 equivalent per g or per ml. (**b**) Fetal demise in USUV-infected mice after cesarean section in the second third of gestation. Spleen (**c**), placenta (**d**), and mammary glands (**e**) of infected pregnant mice were collected and viral burden was measured by RT-qPCR assay. Organs were harvested at E6 and E12 stages of gestation for spleen and placenta, and 2 weeks after delivery for mammary glands.

The percentage of USUV RNA-positive pups’ brains was not significantly different between natural delivery (12%) and cesarean section (6%), while around 16% of naturally delivered pups presented USUV RNA-positive blood two weeks after delivery (Table 1; Figure 5a). Fetal demise was observed in 15% of births after natural delivery, and in 3% of births after cesarean section one week after infection (Table 1; Figure 5b). Differential vertical transmission during the first or second week of gestation could be due to variation in maternal infection. For this reason, we assessed the levels of USUV in the spleen of the pregnant dams one week after infection, in the two conditions of infection. Maternal viral burden in the spleen was not substantially different seven days after inoculation at E6 or E12, suggesting that differences in systemic infection of the pregnant mice are not responsible for the observed phenotype (Figure 5c).

To determine whether direct infection of the placenta occurred, we measured USUV vRNA in the placenta of infected pregnant mice on E6 and E12, and sacrificed them seven days post-infection. We detected viral infection in about half of the placentas tested at E6 and E12. USUV appeared to replicate at higher titers at seven days post-infection (98-fold, *p* < 0.005) in placentas from dams infected at E6 compared to those infected at E12 (Figure 5d). To determine the possibility of USUV transmission during breastfeeding, because we still detected virus in the blood of pups two weeks after delivery, we sampled the mammary glands of the dams for the presence of the virus in these organs, and found no positive results (Figure 5e).

Overall, these results in mice confirm the data obtained on cells and human placenta, and indicate that USUV, similar to other flaviviruses, can be potentially transmitted by intrauterine route and can induce fetal demise and central nervous system infection at low-level rates. In addition, the gestational stage of the fetus has an impact on the extent of USUV replication in the fetus.

## 4. Discussion

Several studies suggest that several neurotropic flaviviruses, including ZIKV, Japanese encephalitis virus (JEV), St. Louis encephalitis virus (SLEV), and WNV, can cause fetal disease, with congenital infection, and could be involved in pregnancy complications and congenital malformation more frequently than is actually detected [45,46,47,48,49,50,51,52,53,54,55,56,57]. For this reason, we chose to study the ability of the USUV, closely related to WNV, to be transmitted vertically, which had never been investigated until now.

Because of the limited availability of fresh primary placental tissues, human immortalized placental cell lines provide tools for first-intention functional studies. We therefore chose to first examine the permissiveness of USUV in two different placental cell lines: the JAR choriocarcinoma cells [42,43], and the HIPECs [37]. Both cell lines display various key characteristics of human placental trophoblasts [58,59], but with some differences, the JAR possessing the ability to differentiate into syncytiotrophoblastic cells in vitro, and the HIPECs corresponding to extravillous cytotrophoblasts with invasive capacities. Both JAR cells and HIPECs were permissive for USUV, albeit with different efficiencies. JAR cells were more efficiently infected than HIPECs, with higher mortality rate, and higher production of infectious viral particles than HIPECs. Using a RT-qPCR multi-array device, we observed a strong antiviral response in JAR cells, characterized by the induction of some PRRs, interferon pathway molecules, ISGs, and chemokines such as CXCL10, CCL5, and CCL11. This pattern of gene induction was similar as what was observed in other cell types upon USUV infection, such as astrocytes [60]. In contrast, HIPECs were not able to elicit a strong antiviral immune response against USUV, with few induced genes and a very low level of induction. Hence, these data may reflect the variety of virus–cell interactions in a multicellular organ such as placenta, where closely related cell types organized in a defined cytoarchitecture, may be differentially permissive to USUV and elicit variable innate antiviral responses.

In a second experiment, we used primary explants of human placenta, from first- and third-trimester, to examine whether human placenta could allow productive replication of USUV, depending on gestational age. At the end of the two-week histoculture, placental tissues were positive for USUV vRNA (and for Env protein for some of them), indicating that USUV was able to express viral transcripts along the duration of the culture. Moreover, even if the quantities were low and variable between placentas, infectious particles were released from tissues explants, albeit with an apparent slightly better efficiency for third-trimester rather than first-trimester placenta. These quantities, although less important than what can be observed from ZIKV infected placental histocultures [32,61], nevertheless suggest that human placenta may be permissive for USUV viral replication. However, it should be kept in mind that JAR and HIPEC cell lines as well as placental histocultures do not perfectly reflect the pathophysiology of the infection, in particular because they lack the adaptive immunity component, which cannot be explored in these models.

Finally, our experiments suggest that USUV can be potentially transmitted by the intrauterine route in immunocompetent mice and induce fetal demise when infection occurred at the first week of pregnancy. With regard to *Flaviviruses*, the most striking effect of congenital infection has been described for ZIKV. The congenital Zika syndrome, consisting in microcephaly and other neurodevelopmental defects, is probably linked to the ability of ZIKV to replicate in the placenta and cross the blood–placental barrier [62]. ZIKV infection in mice during early pregnancy resulted in placental insufficiency and fetal demise whereas infections at late pregnancy caused no apparent fetal disease [36,45]. Vertical transmission also has been demonstrated for WNV in a mice model, showing that the virus is efficiently transmitted by vertical routes (intrauterine and lactation) even at the third week of pregnancy [24]. For both ZIKV and WNV, the vertical transmission rates are much more important than for USUV. For example, after natural delivery, 80% of fetuses from dams infected by WNV at the second week of pregnancy exhibit RNA virus in their brains [24], whereas for USUV, this figure is only 12% after the first week, and none after the second week. Moreover, fetal demise was detected in approximately 50% of WNV-infected animals [23]. Since we still detect viral load in blood of suckling mice two weeks after delivery it would be interesting to study the long-term impact on surviving pups after infection of pregnant females.

In our study, we observed a quite similar permissiveness of the human placenta to infection in first- and third-trimester placenta ex vivo, but vertical transmission in mice was observed following infection in the first tier of gestation only. During the ZIKV epidemic, observational data showed that ZIKV-associated congenital microcephaly was most common when pregnant women were infected during the first or early second trimesters of pregnancy [63]. Moreover, studies in mice described that the placenta and fetus were more susceptible to ZIKV infection at earlier gestational stages [36]. In Ifnar1^−/−^ mice, ZIKV infection at embryonic day six (E6) resulted in fetal demise, infections at mid-stage (E9) resulted in fetal morphologic abnormalities, and infection later in pregnancy (E12) caused no apparent fetal disease [36]. The placenta is a physical and immunological barrier that undergoes important changes during gestation, particularly between the beginning (first trimester) and the end (second and third trimesters) of human pregnancy [64]. The mechanisms underlying the gestational-stage-dependent variation in fetal injury following ZIKV infection have not been fully elucidated. The reduced susceptibility to ZIKV or USUV infection at later stages of gestation could result from differential spatio-temporal expression patterns of putative entry receptors, as suggested for ZIKV [31,32]. Alternatively, and additionally, early and late placenta could be distinguished by their innate immune profiles, notably by their type I and III interferon response [34,36,65,66] or by the expression level of the primate- and placenta-specific C19MC miRNA cluster, known to exert an antiviral activity, and the expression of which are temporally regulated during pregnancy [67,68,69]. Moreover, the fact that human and murine gestation and immune system are very different has to be taken into consideration, and results obtained in mice cannot be directly translated to humans.

In conclusion, our observations suggest that the emergence of the USUV in the human population could potentially represent a subject of concern in pregnant women, since the virus could be vertically transmitted to the fetus in a sporadic manner. Further studies are needed to better characterize the potential for vertical transmission of USUV, and to elucidate the capacity of this virus to cross the blood–placental barrier.

## Figures and Tables

**Table 1:** USUV-positive brains and blood from babies born from experimentally infected mothers. Viral RNA was detected by quantitative RT-qPCR targeting the USUV NS5 gene.

Week of Pregnancy	Type of Birth	% of RT-PCR Positive Brain	% of RT-PCR Positive Blood	% of Death/Birth Defect
First	Natural delivery	12%	16% (2 weeks after delivery)	15% (11/73) *
	Cesarean (2° week)	6%	ND	3% (2/52) #
Second	Natural delivery	0%	0%	0% (0/41)

* Death/birth defect observed in 3 different littermates among the 9 dams infected. # Birth defect observed in 1 littermate among 6 dams infected. ND: Not determined.

## Data Availability

Not applicable.
